# ‘Setting the Benchmark’ Part 2: Contextualising the Physical Demands of Teams in the FIFA World Cup Qatar 2022

**DOI:** 10.5114/biolsport.2024.131091

**Published:** 2023-09-07

**Authors:** Paul S. Bradley

**Affiliations:** 1 FIFA, Zürich, Switzerland

**Keywords:** Match Analysis, High-Intensity, International Football, Playing Position, Soccer

## Abstract

This study aimed to contextualise and benchmark the physical demands of teams in the FIFA World Cup Qatar 2022. With FIFA’s official approval, all sixty-four games were analysed during the competition (*n* = 32 teams) using a multi-camera computerised tracking system. On average, teams during Qatar 2022 covered around 108.1 ± 3.6 km in total, with 9.0 ± 0.9 and 2.3 ± 0.3 km covered at the higher intensities (≥20.0 and ≥25.0 km · h^-1^), respectively. Compared to the FIFA World Cup Russia 2018, national teams in Qatar 2022 covered only 3% more total distance but 16–19% more distance at the higher intensities (*P* < 0.01; Effect Size [ES]: 0.9–2.0). When the data was adjusted based on the number of minutes played, tournament differences at the higher intensities were less pronounced (9–12%; *P* < 0.01; ES: 0.7–1.3). The United States, Canada, Saudi Arabia, Germany and IR Iran covered 19–34% more high-intensity distance than Argentina, Ecuador, Qatar, Poland and Costa Rica during the 2022 tournament (*P* < 0.01; ES: 3.2–3.5). Match-to-match variation of each team in Qatar 2022 revealed Ecuador and Uruguay were particularly consistent for the distances covered at higher intensities (Coefficient of Variation [CV]: 2–3%), whilst Japan demonstrated considerable variation (CV: 23–29%). Teams generally covered more total distance on a per-minute basis in the first versus the second half (*P* < 0.01; ES: 1.2), but no differences existed at higher intensities (*P* > 0.05; ES: 0.0–0.1). Correlations between the number of high-intensity runs and various phase of play events across all teams were strongest for defensive transitions and recoveries, in addition to progressions up the pitch and into the final third (*r* = 0.63–0.75; *P* < 0.01). The present findings provide valuable context into the contemporary team demands of international football. This information could be useful for practitioners to benchmark team performances and to potentially understand the myriad of factors impacting physical performances.

## INTRODUCTION

The highly changeable nature of football results in players continually alternating between brief bouts of high-intensity running and longer periods of low-intensity activity [1]. Researchers have extensively examined this activity profile but primarily from an individual and positional point of view [2–4], as opposed to a team perspective. Football is a team sport whereby the activities of players are mutually dependent upon the actions of their teammates and the opponent [5–6]. Thus, this necessitates the need for more research on team physical performances, particularly on the myriad of factors that up or down regulate physical outputs. Although some team physical benchmarking has occurred for various domestic leagues [7–9], scant evidence exists for international competitions such as the FIFA World Cup Qatar 2022. This information would certainly serve as an important point of reference for practitioners regarding the contemporary team demands of international football.

Football is an ever-changing game with research highlighting that the physical match demands have evolved significantly in the last decade, especially from a high-intensity perspective [10]. This type of evolution has been found in the English Premier League [3], Spanish La-Liga [11], Chinese Super League [9], and the FIFA Women’s World Cup [12]. Despite this, no published work has established if this trend exists at recent international tournaments (e.g., FIFA World Cup Russia 2018 versus Qatar 2022). This is particularly relevant given new directives employed in Qatar 2022 that increased the number of substitutes compared to Russia 2018 [13]. This rule modification may contribute to even greater evolutionary changes from a team physical perspective (e.g., moving from three to five substitutes). Moreover, new directives to account for all time loss activities in the FIFA World Cup Qatar 2022 [14], resulted in much longer second halves than previous tournaments. Consequently, the team distances covered across halves in total and at higher intensities may have been modified and this warrants further investigation.

A powerful modulating factor influencing a team’s physical output is the tactical approach that they employ [15]. A major challenge for practitioners is synchronising the physical and tactical metrics together to determine this. This is accomplished through simultaneously aligning the physical efforts with the tactical phases of play or scenarios [16–20]. Unfortunately, this type of integration can be immensely complex, so some authors simply determine noteworthy associations between physical-tactical-technical metrics [21]. Thus, correlating the physical data with FIFA’s Enhanced Football Intelligence metrics may provide much-needed context as to why teams physically exerted themselves during FIFA World Cup matches. Therefore, this study aimed to contextualise and benchmark the team demands during the FIFA World Cup Qatar 2022.

## MATERIALS AND METHODS

### Sample

With FIFA’s official approval, all games during the FIFA World Cup Qatar 2022 were collected and analysed. Team analyses involved the summation of all match physical performance values of outfield players who participated in games, including substitutes (goalkeeper data excluded). Thus, data trends are the sum of all individual outfield player values presented as team totals. Team data consisted of all 32 nations across 64 game observations. As this data are freely available [22], no ethical approval was required.

### Match Analysis System

All FIFA World Cup Qatar 2022 games were analysed using a multicamera computerised tracking system (TRACAB, Chyron Hego). All player movements were captured by high-definition cameras operating at 25 Hz. This systems validity has been quantified to verify the capture process and subsequent accuracy of the data [23]. After system calibration and various stringent quality control processes, the data captured were analysed using match analysis software. This produced a data set on each team’s activity patterns during a match using specified speed zones.

### Speed Zones

Players’ activities were coded into the following:

- Zone 1 (0.0–6.9 km · h^-1^),
- Zone 2 (≥7.0–14.9 km · h^-1^),
- Zone 3 (≥15.0–19.9 km · h^-1^),
- Zone 4 (≥20–24.9 km · h^-1^),
- Zone 5 (≥25.0 km · h^-1^).

Total distance represented the sum of the distances covered above. High-intensity activity consisted of the aggregation of Zones 4 and 5 (≥20.0 km · h^-1^), whilst sprinting exclusively included Zone 5 activity (≥25.0 km · h^-1^). Similar classifications for the upper two Zones have been employed in elite football for over a decade [24]. Moreover, the speed demarcations used for the FIFA World Cup Qatar 2022 were identical to those employed at the FIFA World Cup Russia 2018 [25], thus allowing tournament comparisons to occur.

### Enhanced Football Intelligence Metrics

To further contextualise the physical trends, FIFA’s Enhanced Football Intelligence metrics were also adopted [26], specifically the phases of play metrics that captured the tactical behaviours of teams during games. FIFA’s algorithms quantified different in-possession phases (build up, progression, final third, attacking transition, counter-attack, long ball and set piece) and out-of-possession phases (low, mid, high block/press, counter-press, recovery and defensive transition) using the tracking data obtained from the previously described system. It used various features (spatial and physical) to identify and classify the various phases of play. For instance, it extracted ball and player pitch locations in relation to each other, in addition to the speed and direction of play. If teams entered a certain phase of play for a selected period of time, then the algorithms recorded this as a frequency count or an accumulated fraction of in-possession or out-of-possession time. Detailed definitions for all in- and out-of-possession phases of play can be found in freely available documentation [26].

### Statistical Analyses

All statistical analyses were conducted using SPSS (SPSS Inc., Chicago, USA). Descriptive statistics were calculated on each variable and z-scores were used to verify normality. Performance differences across teams and halves were determined using paired-samples and independent t-tests. Statistical significance was set at *P* < 0.05. The coefficient of variation (CV) was calculated to determine the data spread across each metric. Effect sizes (ES) were computed to determine the meaningfulness of any differences and corrected for bias using Hedges formula. The ES magnitudes were classed as trivial (< 0.2), small (> 0.2–0.6), moderate (> 0.6–1.2) and large (> 1.2). Pearson’s coefficients were used for correlation analyses and the magnitudes of the associations were regarded as trivial (*r* ≤ 0.1), small (*r* > 0.1–0.3), moderate (*r* > 0.3–0.5), large (*r* > 0.5–0.7), very large (*r* > 0.7–0.9), and nearly perfect (*r* > 0.9). Values are presented as means and standard deviations unless otherwise stated.

## RESULTS

### Benchmarking & Match-to-Match Variation

On average, teams during the FIFA World Cup Qatar 2022 covered 108.1 ± 3.6 km in total, with 9,001 ± 850 m and 2,345 ± 314 m covered at the higher intensities (≥20.0 and ≥25.0 km · h^-1^), respectively. The physical performances of teams at the upper and lower extremes also revealed some interesting trends. Figure 1A demonstrates that the top five ranked teams for total distance (United States, IR Iran, Australia, Canada, Serbia) covered 8–14% more in games compared to the bottom five ranked teams (Qatar, Brazil, Argentina, Mexico, Ecuador) during the FIFA World Cup Qatar 2022 (*P* < 0.01; ES: 2.0–4.5). In Figure 1B, it is noteworthy that the top five ranked teams from a high-intensity running perspective (United States, Canada, Saudi Arabia, Germany, IR Iran) covered 19–34% more distance than the bottom five ranked teams (Argentina, Ecuador, Qatar, Poland, Costa Rica) during the competition (*P* < 0.01; ES: 3.2–3.5). Figure 1C illustrates that the top five ranked sprinting teams (United States, Saudi Arabia, Canada, Uruguay, Germany) covered 21–49% more distance than the bottom five ranked teams (Japan, Denmark, Netherlands, Australia, Costa Rica) during the tournament (*P* < 0.01; ES: 1.2–3.0).

**FIG. 1A:**
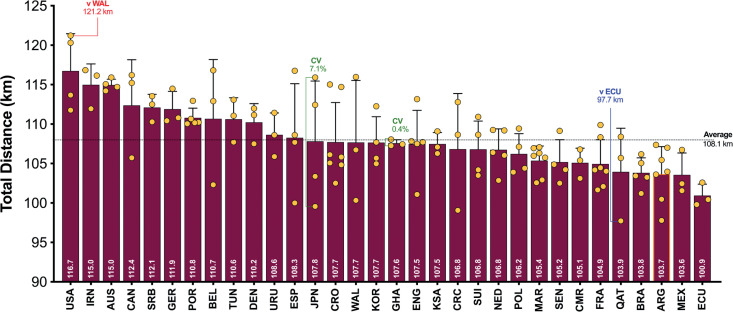
Total Team Distance and match-to-match variation in the Qatar FIFA World Cup 2022. Data normalized for 90+ min (excludes GK and extra time). Red = max, Blue = min, Green = variation.

**FIG. 1B:**
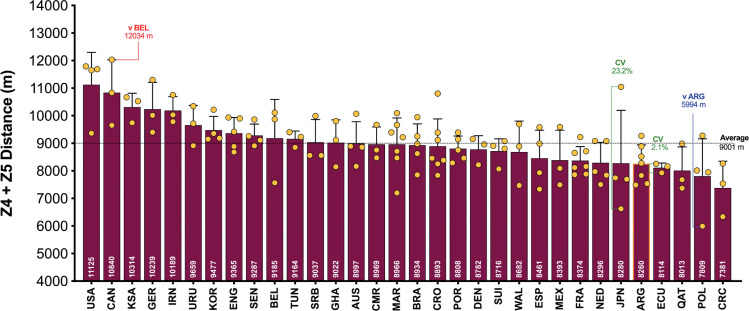
Team High Intensity Distance (≥20 km · h^-1^; Zone 4 and 5; Z4+Z5) and match-to-match variation in the Qatar FIFA World Cup 2022. Data normalized for 90+ min (excludes GK and extra time). Red = max, Blue = min, Green = variation.

**FIG. 1C:**
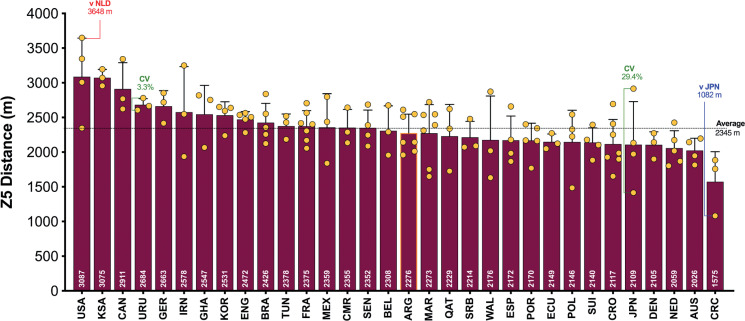
Team Sprint Distance (≥25 km · h^-1^; Zone 5; Z5) and match-to-match variation in the Qatar FIFA World Cup 2022. Data normalized for 90+ min (excludes GK and extra time). Red = max, Blue = min, Green = variation.

Figures 1A–C also depict the match-to-match variation of each team in the FIFA World Cup Qatar 2022. On average, teams match-to-match CV’s during the tournament for total and the distance covered at higher intensities (≥20.0 and ≥25.0 km · h^-1^) were 3.2%, 9.1% and 13.9%, respectively. The most consistent team from a physical perspective was highly dependent on the metric. For instance, Ghana, Ecuador and Uruguay were particularly consistent for total distance (CV: 0.4%), high-intensity distance (CV: 2.1%) and sprint distance (CV: 3.3%), respectively. It is noteworthy that Japan exhibited the most variation from match-to-match for the distance covered in total (CV: 7.1%) and that covered at higher intensities (CV: 23.2–29.4%).

### Quadrant Plots

The data presented in Figures 2A–B correlates two distinct dimensions of physical performance using quadrant plots to compare each team against one another. Figure 2A demonstrates a large association between a team’s total and high-intensity game distances (*r* = 0.65; *P* < 0.01). The distribution of teams in each quadrant indicated that ~50% were in the lower-left quadrant (Ecuador, Costa Rica, Qatar, Poland, Argentina, France, Netherlands, Japan, Wales, Croatia, Ghana, Switzerland, Morocco, Cameron, Brazil, Mexico), ~16% were in the lower-right quadrant (Serbia, Australia, Portugal, Denmark, Spain), ~13% were in the upper-left quadrant (Saudi Arabia, Korea Republic, England, Senegal) and ~22% were in the upper-right quadrant (United States, Canada, Germany, IR Iran, Belgium, Tunisia, Uruguay). Figure 2B demonstrates a moderate association between a team’s total and sprint game distances (*r* = 0.33; *P* > 0.05). The distribution of teams in each quadrant indicated that ~34% were in the lower-left quadrant (Costa Rica, Ecuador, Argentina, Qatar, Morocco, Switzerland, Poland, Wales, Croatia, Japan, Netherlands), ~19% were in the lower-right quadrant (Australia, Serbia, Portugal, Belgium, Denmark, Spain), ~28% were in the upper-left quadrant (Saudi Arabia, Ghana, Korea Republic, England, Cameron, France, Brazil, Mexico, Senegal) and ~19% were in the upper-right quadrant (Uruguay, Tunisia, Germany, Canada, IR Iran, United States).

**FIG. 2A:**
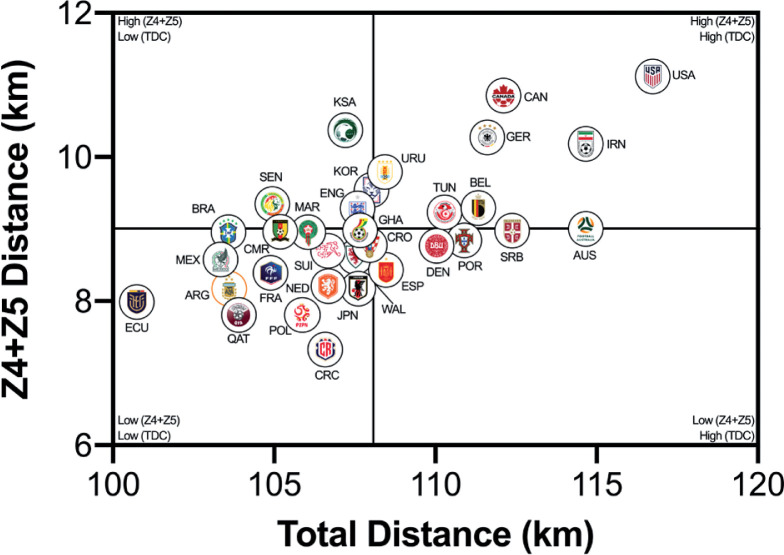
Team Total versus High Intensity Distance (≥20 km · h^-1^; Zone 4 and 5; Z4+Z5) in the Qatar FIFA World Cup 2022. Data normalized for 90+ min (excludes GK and extra time). Crosshairs were based on the average for all teams.

**FIG. 2B:**
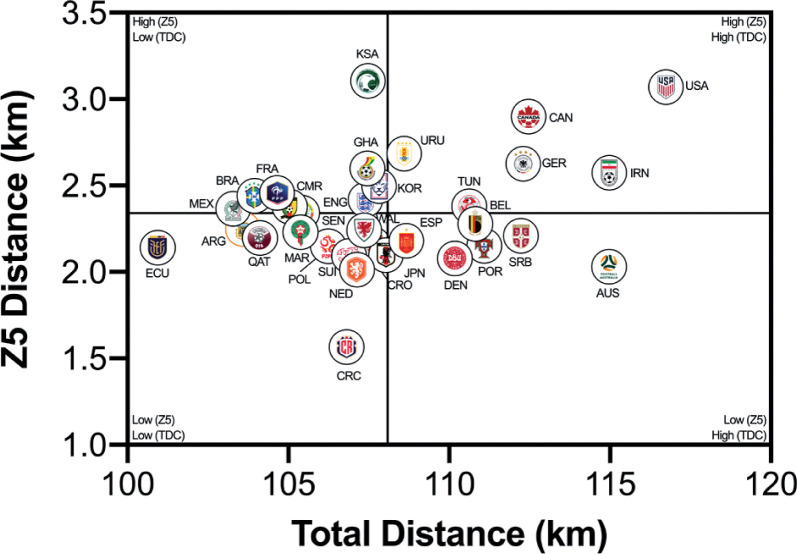
Team Total versus Sprint Distance (≥25 km · h^-1^; Zone 5; Z5) in the Qatar FIFA World Cup 2022. Data normalized for 90+ min (excludes GK and extra time). Crosshairs were based on the average for all teams.

### Physical Evolution

Figures 3A–C demonstrate teams covered only 3% more total distance in the FIFA World Cup Qatar 2022 than in Russia 2018 (*P* < 0.01; ES: 0.9). However, the distances at higher intensities (≥20.0 and ≥25.0 km · h^-1^) were 16–19% greater in Qatar 2022 than Russia 2018 (*P* < 0.01; ES: 1.2–2.0). When the data was adjusted based on the number of minutes played in each tournament, the trends for the overall distance covered actually reversed (3% lower in Qatar 2022 versus Russia 2018; *P* < 0.01; ES: 0.8). Although the demands were still greater in Qatar 2022 for the distances covered at higher intensities, they were less pronounced when adjusted for minutes played (9–12% higher in Qatar 2022 versus Russia 2018; *P* < 0.01; ES: 0.7–1.3).

**FIG. 3A:**
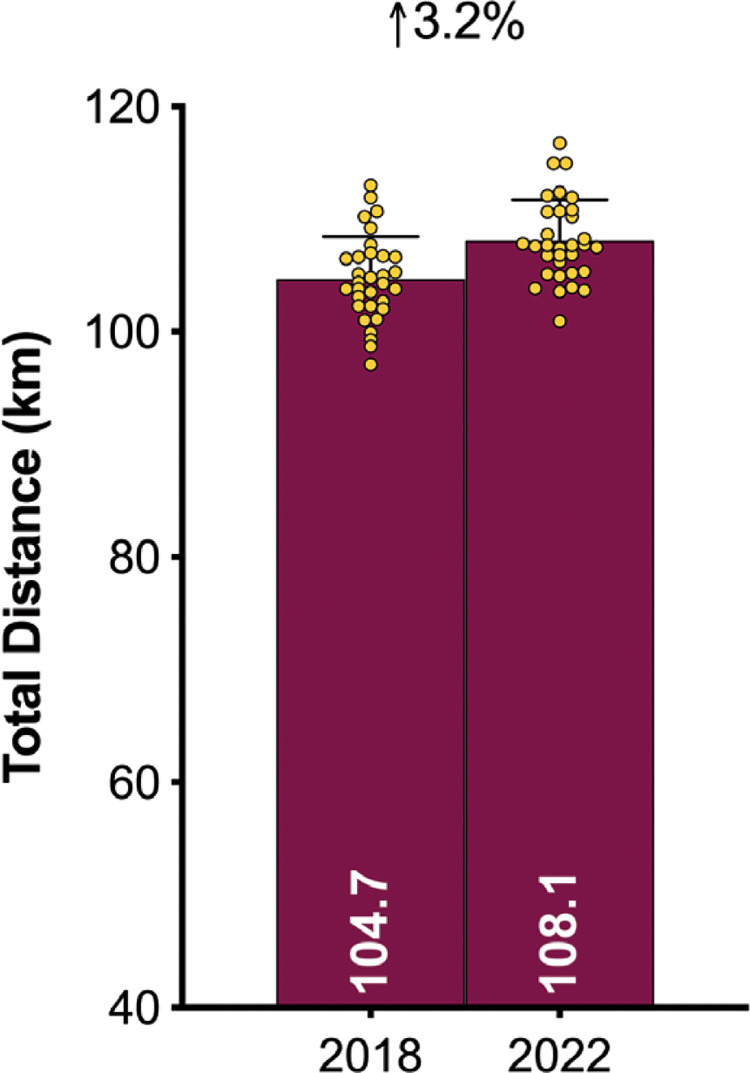
Evolution of the physical demands between FIFA World Cup 2018 vs 2022: Total Distance

**FIG. 3B:**
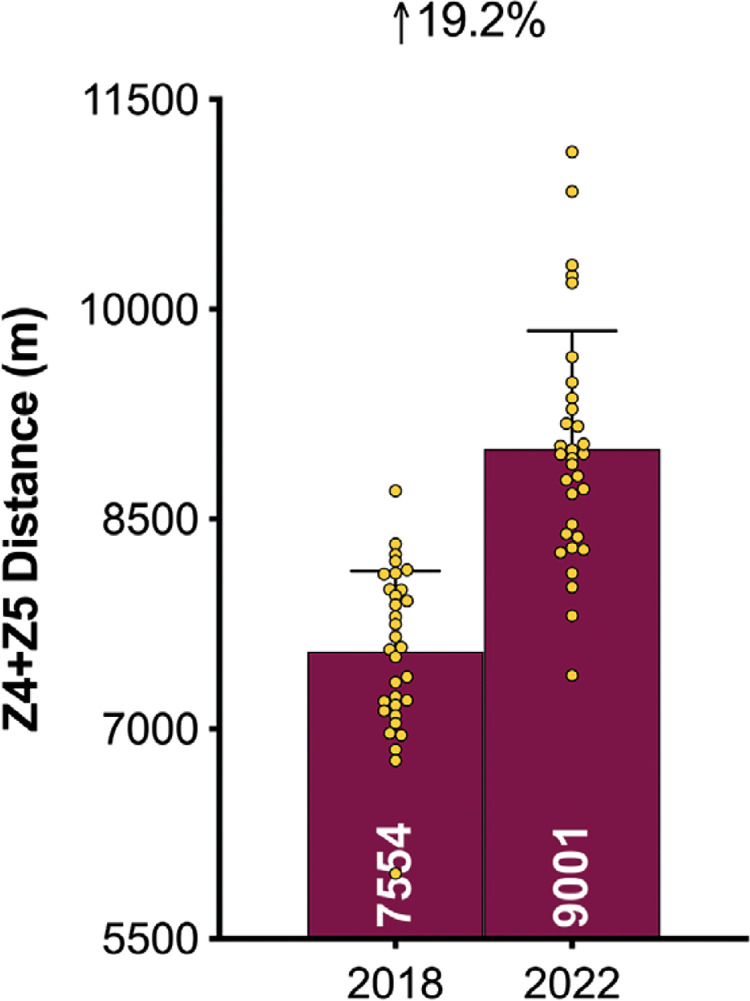
Evolution of the physical demands between FIFA World Cup 2018 vs 2022: High-Intensity Distance (≥20 km · h^-1^; Zone 4 and 5; Z4+Z5).

**FIG. 3C:**
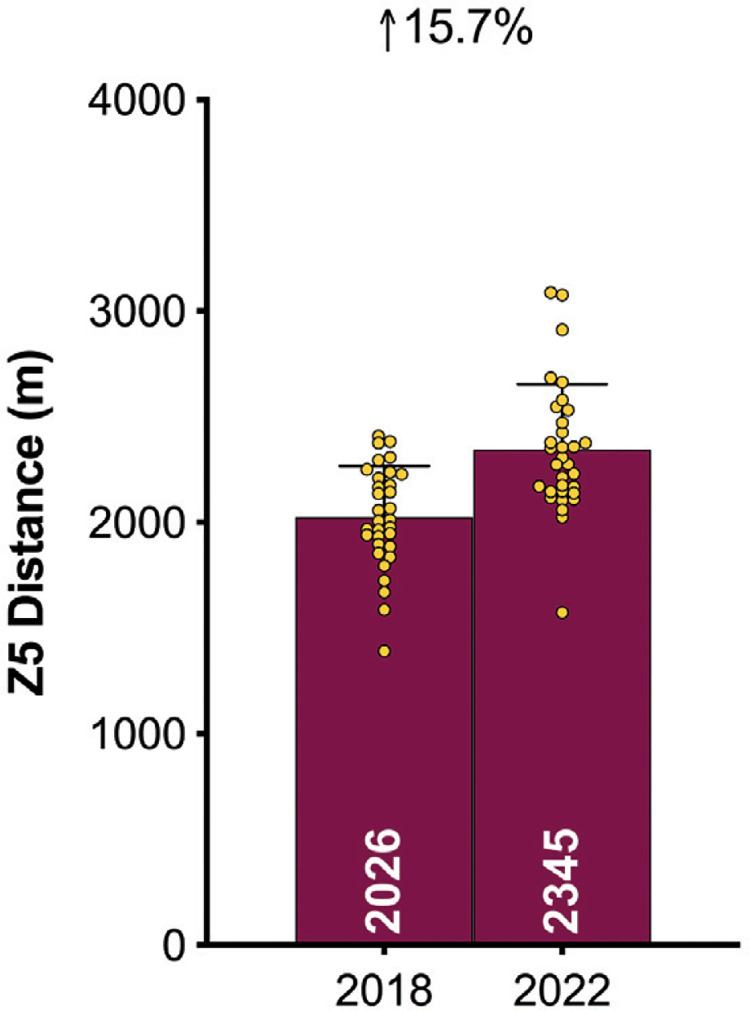
Evolution of the physical demands between FIFA World Cup 2018 vs 2022: Sprinting Distance (≥25 km · h^-1^; Zone 5; Z5).

### Half-by-Half Differences

Figure 4A–C highlighted teams generally covered less total distance on a per-minute basis in the second half than in the first half (*P* < 0.01; ES: 1.2). Although Ecuador, Mexico, Qatar, Cameron and Canada covered similar distances across halves, more pronounced half-by-half differences were evident for Wales, France and Costa Rica. However, no half-by-half deficits existed for the distance on a per-minute basis at higher intensities (≥20.0 and ≥25.0 km · h^-1^) in the FIFA World Cup Qatar 2022 (*P* > 0.05; ES: 0.0–0.1). Outliers such as Wales and Portugal covered much more distance at the higher intensities in the second half of matches, whilst Senegal and Korea Republic covered much more in the first half of matches.

**FIG. 4A:**
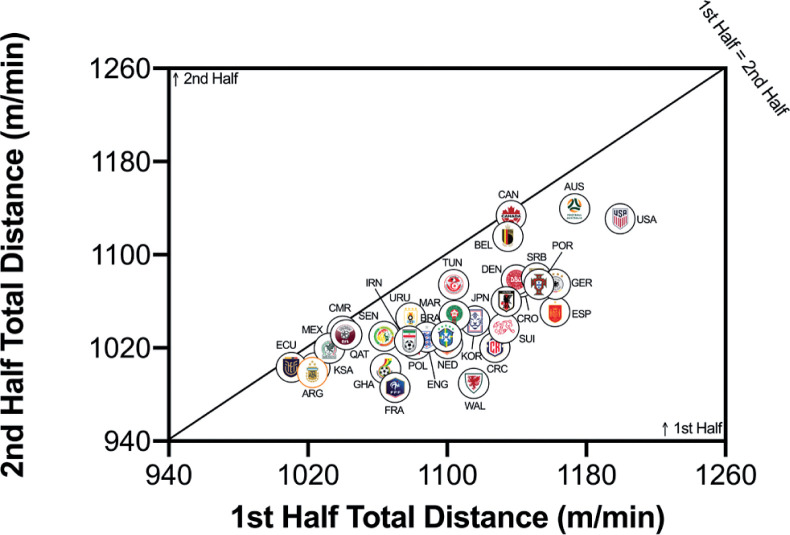
Team half by half Total Distance in the Qatar FIFA World Cup 2022. Data normalized per min and for 90+ min (excludes GK and extra time). Teams on the centre line covered the same distances in the 1st and 2nd half. Teams in the bottom triangle cover more distance in the 1st half and teams in top triangle cover more in the 2nd half.

**FIG. 4B:**
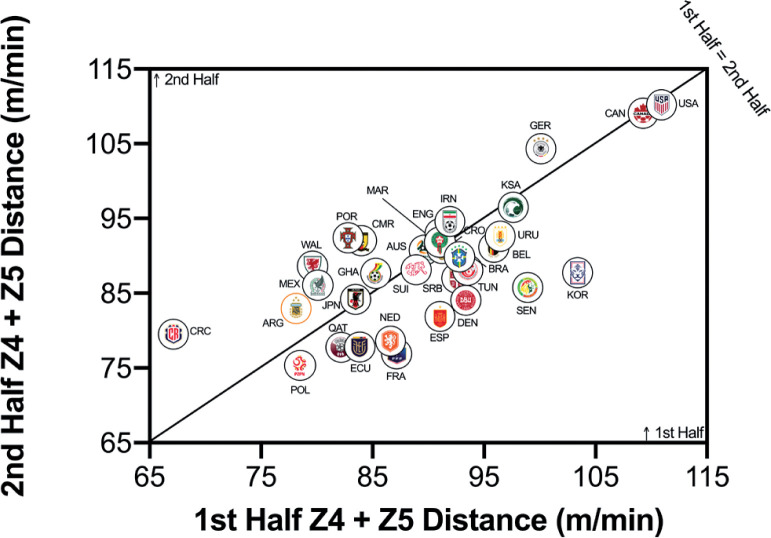
Team half by half High Intensity Distance (≥20 km · h^-1^; Zone 4 and 5; Z4+Z5) in the Qatar FIFA World Cup 2022. Data normalized per min and 90+ min (excludes GK and extra time). Teams on the centre line covered the same distances in the 1st and 2nd half. Teams in the bottom triangle cover more distance in the 1st half and teams in top triangle cover more in the 2nd half.

**FIG. 4C:**
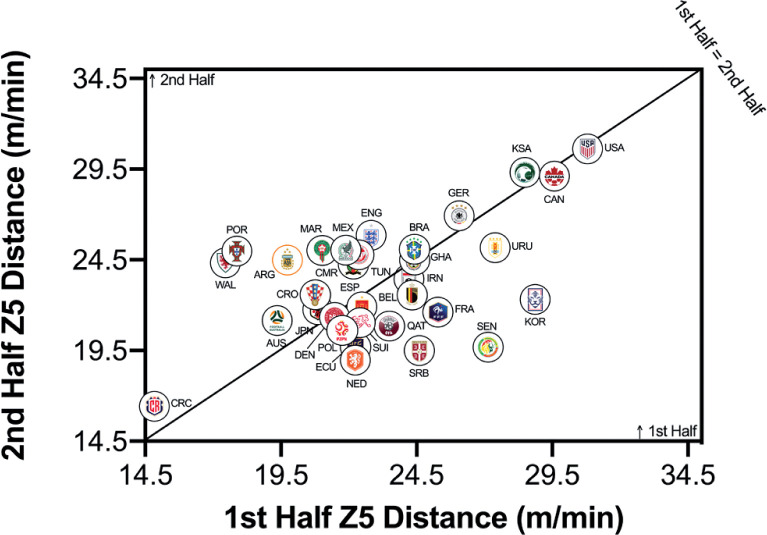
Team half by half Sprint Distance (≥25 km · h^-1^; Zone 5; Z5) in the Qatar FIFA World Cup 2022. Data normalized per min and only players completing 90+ min (excludes GK and extra time). Teams on the centre line covered the same distances in the 1st and 2nd half. Teams in the bottom triangle cover more distance in the 1st half and teams in top triangle cover more in the 2nd half.

### Correlations Between Physical and Tactical Metrics

The number of high-intensity runs across teams were correlated against FIFA’s Enhanced Football Intelligence metrics to determine any noteworthy associations (e.g., *r* > 0.60). Figure 5A indicates a large correlation between the number of high-intensity runs out-of-possession versus the combined number of events for defensive recoveries and transitions (*r* = 0.63; *P* < 0.01). Teams such as the United States, Saudi Arabia, Canada, IR Iran and Australia clearly fall within the upper-right quadrant as they performed a plentiful number of each. Figures 5B and 5C show a very large association between the number of high-intensity runs performed in-possession and the number of progression and final-third events (*r* = 0.73–0.75; *P* < 0.01). Teams such as the United States, Germany, Spain and Brazil were found in the upper-right quadrant, whilst Costa Rica, Poland, Australia and Japan are in the lower-left quadrant for both metrics.

**FIG. 5A:**
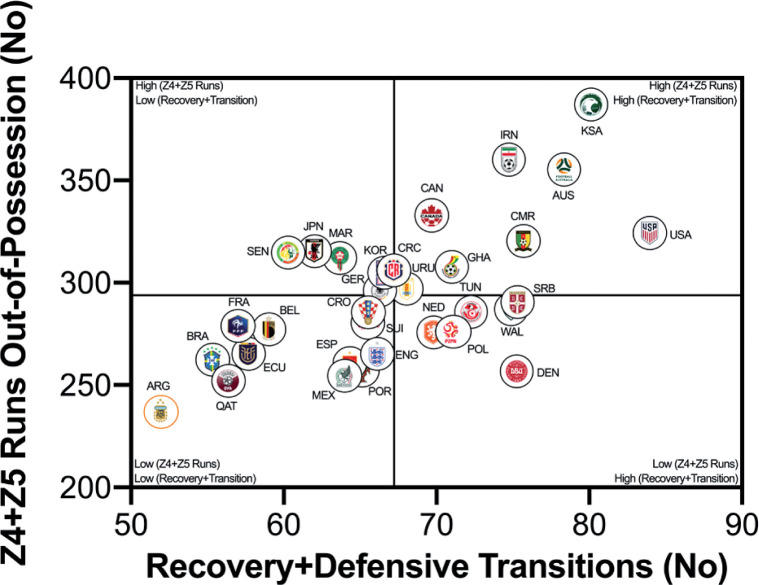
Team Recovery and Defensive Transition Count versus Out of Possession High Intensity Runs (≥20 km · h^-1^; Zone 4 and 5; Z4+Z5) in the Qatar FIFA World Cup 2022. Data normalized for 90+ min (excludes GK and extra time). Correlation; *r*=0.63; *P*<0.01.

**FIG. 5B:**
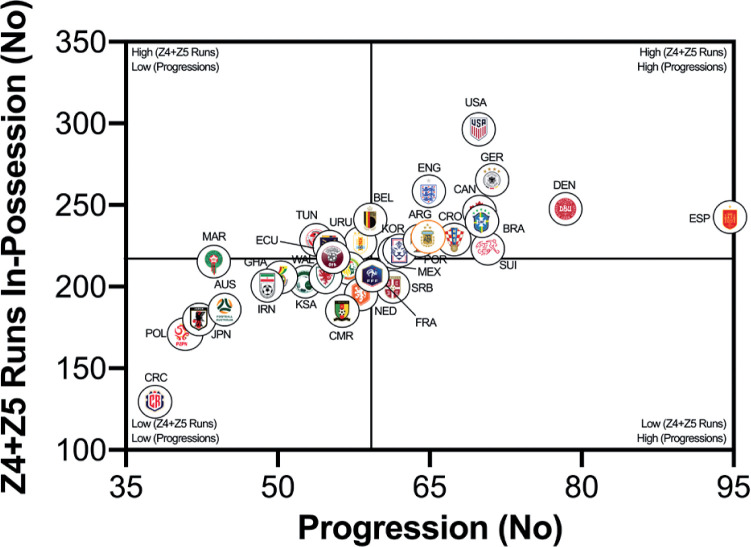
Team Progression Count versus In Possession Runs (≥20 km · h^-1^; Zone 4 and 5; Z4+Z5) in the Qatar FIFA World Cup 2022. Data normalized for 90+ min (excludes GK and extra time). Correlation; *r*=0.73; *P*<0.01

**FIG. 5C:**
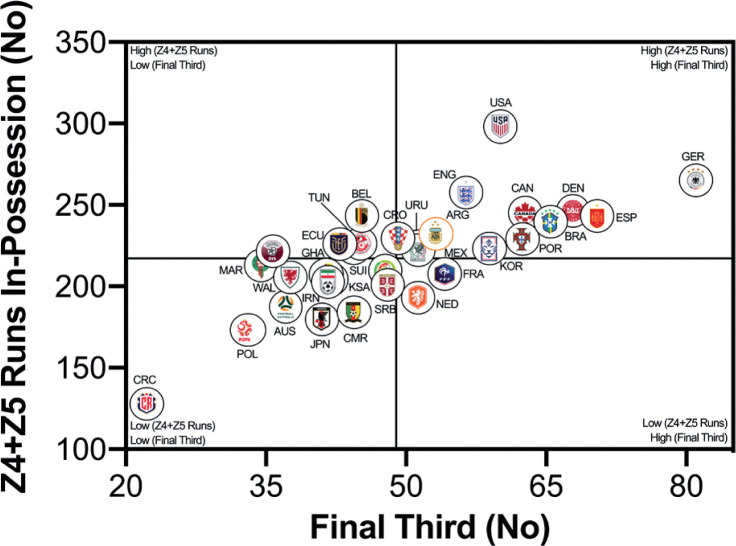
Team Final Third Count versus In Possession Runs (≥20 km · h^-1^; Zone 4 and 5; Z4+Z5) in the Qatar FIFA World Cup 2022. Data normalized for 90+ min (excludes GK and extra time). Correlation; *r*=0.75; *P*<0.01.

## DISCUSSION

This study was the first to physically benchmark all national teams competing at the FIFA World Cup Qatar 2022. On average, teams during the tournament covered around 108 km in total, with 9.0 and 2.3 km covered at the higher intensities (≥20.0 and ≥25.0 km · h^-1^), respectively. Comparative team benchmarks from the FIFA World Cup Russia 2018 [25], revealed that national teams in Qatar 2022 covered only 3% more total distance but around 16–19% more distance at the higher intensities. Although it is tempting to attribute this finding to elevated demands in contemporary international football, the reader should be cognisant of the complexities surrounding such comparisons. For instance, new directives for added time in Qatar 2022 [14], resulted in much longer game durations compared with Russia 2018. However, when the data was adjusted based on the number of minutes played, tournament differences were less pronounced (9–12% at higher intensities). New directives also allowed teams to make five substitutes in Qatar 2022, as opposed to three in Russia 2018 [13]. This could also have contributed to the greater team demands in Qatar 2022, as substitutes cover more distance on a per minute basis at higher intensities compared to those starting the game or those that were replaced [1, 27]. More second half substitutions may also account for the negligible between halve deficits observed for high-intensity metrics in Qatar 2022. Irrespective of the differences in match duration or the number of substitutes used between tournaments, modern international teams are now expected to cover substantial distances at higher intensities. Thus, greater importance should be placed on training modalities that optimally prepare players for the rigours of the modern international game [28–30].

This section will attempt to integrate the present findings with a contextual narrative to aid interpretation. Quadrant plots revealed that teams such as the United States exhibited both volume and intensity characteristics during FIFA World Cup Qatar 2022 matches, whilst the likes of Costa Rica were the antithesis of this. This finding may not necessarily be completely indicative of physical fitness differences, but could also be shaped by the style of play employed by each team and/or their opposition plus numerous other contextual factors [31–32]. This is understandable as the aim of any team’s tactics is to ensure optimal organisation in order to best utilise their physical and technical capabilities [5]. There were some expectations that utilising FIFA’s Enhanced Football Intelligence metrics would shed some light on the tactical factors that up or down regulate a team’s physical exertions during games. Interestingly, the strongest associations between the number of high-intensity runs a team performed and the various phases of play occurred for game situations that had a real urgency attached to their outcome (e.g., risk/benefit). Out-of-possession, this included high-intensity efforts to defensively recover and transition. Due to the potential consequences of not tracking back, it is not surprising that teams work intensely out-of-possession during defensive recoveries and transitions [7, 15–20]. The United States, Saudi Arabia, Canada, IR Iran and Australia clearly resided within the upper-right quadrant as they performed a plentiful number of each. Similarly, in-possession, this included high-intensity efforts to progress quickly up the pitch and into the final third to be an attacking threat. This could suggest that teams like the United States, Germany, England, Spain and Brazil up their intensity once they progress the ball forward and/or into the final third via vertical passes or dribbling. Research has revealed that the greatest proportion of a team’s high-intensity activity occurs during transition-based activities [17, 19–20]. Thus, the United States intensity could be associated with their frequent transitions to recover defensively and to progress offensively, which may require players to produce long linear high-intensity runs. Whilst Costa Rica frequently sat compactly in a defensive low- or mid-block for extended periods, and this may have reduced their opportunity to move into space to engage in high-intensity activities, hence their subdued game intensity.

The present study was the first to quantify the physical match-to-match variation of each team in the FIFA World Cup Qatar 2022. Research has revealed that from an individual/positional perspective, the total distance covered is relatively stable from match-to-match but the distance covered at higher intensities varies considerably [33–34]. The present findings confirm this assertation from a collective perspective as team match-to-match CV’s during the tournament were only 3% for the total distance covered but 9–14% for the distance covered at higher intensities. The most consistent teams from a physical perspective were highly dependent on the metric. For instance, Ghana, Ecuador and Uruguay were particularly consistent for total distance, high-intensity and sprint distances, respectively. It is noteworthy that Japan exhibited the most variation from game to game across most physical metrics. The opposition that Japan played against at the upper and lower ends of the range provides much-needed context. Japan covered their greatest distances in total and at higher intensities against Germany because the duration of that match was much longer than their other games. Japan covered most of this distance while out-of-possession (22% possession) in a reactive attempt to press Germany and force turnovers. In contrast, Japan covered their lowest distances in total and across higher intensities against Costa Rica. Japan covered more distance while in-possession (49% possession) across metrics in this game due to the defensive low/midblock tactics that Costa Rica employed. Thus, Japan was able to dictate play more, as evidenced by their highest number of build-ups, progressions and movements to receive of their tournament. Given the multi-facetted and variable nature of football performance, the identification of factors that up or down regulate physical outputs is incredibly challenging as the context changes considerably within and between games. Thus, practitioners may need to focus their lens on game-by-game trends to gain a more holistic understanding of the impact of contextual influences on team physical outputs.

Although the present study provides unique insights into the physical team demands at the FIFA World Cup Qatar 2022, the reader should be aware of various shortcomings. The physical data presented should ideally include acceleration and change of direction metrics to provide a more rounded overview of team demands. Moreover, presenting the physical data trends across intensified periods of play would have enabled greater translation into training drill formats. Despite identical speed zones adopted across tournaments, the evolutionary trends should be viewed with caution given technological factors could have been modified (e.g., filters used to quantify high-intensity runs). Finally, the direct integration of physical and tactical metrics was not possible for this study. As a correlational approach was adopted, it is important for the reader to be mindful that correlation does not equal causation when examining these trends. The reader should also be cognisant of connecting the dots between team and positional trends by viewing other sources [35], this will provide a more holistic understanding of match demands at the international level.

## CONCLUSIONS

This study was the first to verify the upper and lower physical benchmarks for international teams competing at the FIFA World Cup Qatar 2022. The data demonstrated the high physical demands of contemporary international football, which could be a combination of improved physical preparation and new rule directives applied to this tournament (e.g., more added time and substitutes). Practitioners should be cognisant of the fact that a team’s physical demands are shaped by a myriad of factors and this makes interpretations particularly challenging.
